# Automated detection of smiles as discrete episodes

**DOI:** 10.1111/joor.13378

**Published:** 2022-10-20

**Authors:** Hisham Mohammed, Reginald Kumar, Hamza Bennani, Jamin B. Halberstadt, Mauro Farella

**Affiliations:** ^1^ Discipline of Orthodontics, Faculty of Dentistry University of Otago Dunedin New Zealand; ^2^ School of Information Technology, Otago Polytechnic Dunedin New Zealand; ^3^ Department of Psychology University of Otago Dunedin New Zealand; ^4^ Discipline of Orthodontics, Department of Surgical Sciences University of Cagliari Cagliari Italy

**Keywords:** orthodontics, smiling, validation studies

## Abstract

**Background:**

Patients seeking restorative and orthodontic treatment expect an improvement in their smiles and oral health‐related quality of life. Nonetheless, the qualitative and quantitative characteristics of dynamic smiles are yet to be understood.

**Objective:**

To develop, validate, and introduce open‐access software for automated analysis of smiles in terms of their frequency, genuineness, duration, and intensity.

**Materials and Methods:**

A software script was developed using the Facial Action Coding System (FACS) and artificial intelligence to assess activations of (1) cheek raiser, a marker of smile genuineness; (2) lip corner puller, a marker of smile intensity; and (3) perioral lip muscles, a marker of lips apart. Thirty study participants were asked to view a series of amusing videos. A full‐face video was recorded using a webcam. The onset and cessation of smile episodes were identified by two examiners trained with FACS coding. A Receiver Operating Characteristic (ROC) curve was then used to assess detection accuracy and optimise thresholding. The videos of participants were then analysed off‐line to automatedly assess the features of smiles.

**Results:**

The area under the ROC curve for smile detection was 0.94, with a sensitivity of 82.9% and a specificity of 89.7%. The software correctly identified 90.0% of smile episodes. While watching the amusing videos, study participants smiled 1.6 (±0.8) times per minute.

**Conclusions:**

Features of smiles such as frequency, duration, genuineness, and intensity can be automatedly assessed with an acceptable level of accuracy. The software can be used to investigate the impact of oral conditions and their rehabilitation on smiles.

## 1 INTRODUCTION

Smiling is a spontaneous facial expression occurring throughout everyday life, which varies largely between individuals.^1^ While the interpretation of smiling may appear straightforward, it is actually one of the most complex facial expressions, and can be ambiguous.^2^ Not only can smiles have different forms and meanings, but they are also found in different situations and as a consequence of different eliciting factors.^3^, ^4^

Smile analysis in dentistry has largely focused on static images.^5^ Nonetheless, more recently, there has been a paradigm shift in treatment planning and smile rehabilitation from using static smiles to dynamic smiles; herein lies the ‘art of the smile’.^6^ As the pursuit for better dentofacial aesthetics increases, it is essential to distinguish between posed and spontaneous smiles, differences between which are significant and can influence treatment planning and smile design.^5^ Understanding the characteristics of different smiles and the associated age‐related changes in orofacial musculature, for example, is important to the decision‐making process to achieve ‘ideal’ tooth display.^7^ However, this process should not be confined to the aesthetic elements alone but should also extend to understand whether an oral rehabilitation treatment, including orthodontics, actually affects the number and the way a patient smiles.^8^, ^9^

Smiling that depicts situations of spontaneous pure enjoyment or laughter are often referred to as the genuine ‘Duchenne’ smiles, to acknowledge the scientist who first described their features.^10^, ^11^ The Duchenne smile prompts a combined activation of the *zygomaticus major* and the *orbicularis oculi* muscles. This pattern of muscular activity distinguishes between genuine smiles and ‘social’ smiles, which are generally expressed during conditions of non‐enjoyment.^12^, ^13^ The identification of Duchenne smiles relies on subtle analysis of facial expressions.^14^

The Facial Action Coding System (FACS)^15^ is a popular and reliable method for detecting and quantifying the frequency of facial expressions from full‐face video recordings.^16^ The FACS uses action units (AUs), which code for actions of individual or groups of muscles during facial expression.^15^ The activation level of each AU is scored using intensity scores, ranging from ‘trace’ to ‘maximum’. According to FACS, the onset of a smile can be identified when the activation of the *zygomaticus major* displays traces of raised skin within the lower‐to‐middle nasolabial area and other traces of upwardly angled and elongated lip corners.^15^ These muscle activities would increase in intensity until the smile apex is reached before reverting until no further traces of activation of the *zygomaticus major* could be recognised; hence, the smile offset is denoted.^15^ The introduction of FACS has undoubtedly challenged the study of facial expressions as it allows real‐time assessment of emotions; however, its utilisation for manual detection and coding of AUs presents with limitations; (a) the need for experienced coders who are able to accurately identify on a frame‐wise basis the onset, apex, and offset of a smile,^16^ (b) the coding process is extremely laborious, posing a huge challenge in large‐scale research, (c) susceptibility to observer biases^17^ and high costs.^18^ The observable limitations encountered with manual analyses of smiles has led to computing developments to automatedly detect dynamic smiling features.^19^

FACS focuses primarily on the identification of active target AUs frame‐by‐frame and do not include comprehensive analyses of smiling as discrete episodes, so that their individual features and patterns can be characterised. An episode‐wise analysis of individual smiles would allow researchers to address questions such as how often, how long, how strong, and how genuinely do individuals smile under different experimental and/or situational factors, and what is the impact of factors such as, oral health‐related conditions, on the way people smile. This would also pave the way to understand the dynamic characteristics of smiles in oral rehabilitation patients^8^ and assist in areas where smile rehabilitation through individualised muscle mimicry and training is demanded.^20^

The aim of this study is to develop and validate a user‐friendly software script, based on well‐established pattern‐recognition algorithms for tracking facial landmarks and facial AUs, so that discrete smile episodes can be analysed off‐line from full‐face videos and quantified in terms of frequency, duration, authenticity, and intensity of smile.

## 2 MATERIALS AND METHODS

The study included two phases. During the first phase, a software script was developed with the help of a computer scientist (HB) and extensively tested with ongoing feedback from a focus group represented by the authors and a few test volunteers. During the second phase, preliminary data were collected from a convenience sample of thirty study participants to optimise the performance of the algorithm for smiling detection and to identify optimal thresholds, so that the software's performance could be validated against two manual coders.

### 2.1 Phase I: software script

**FIGURE 1:**
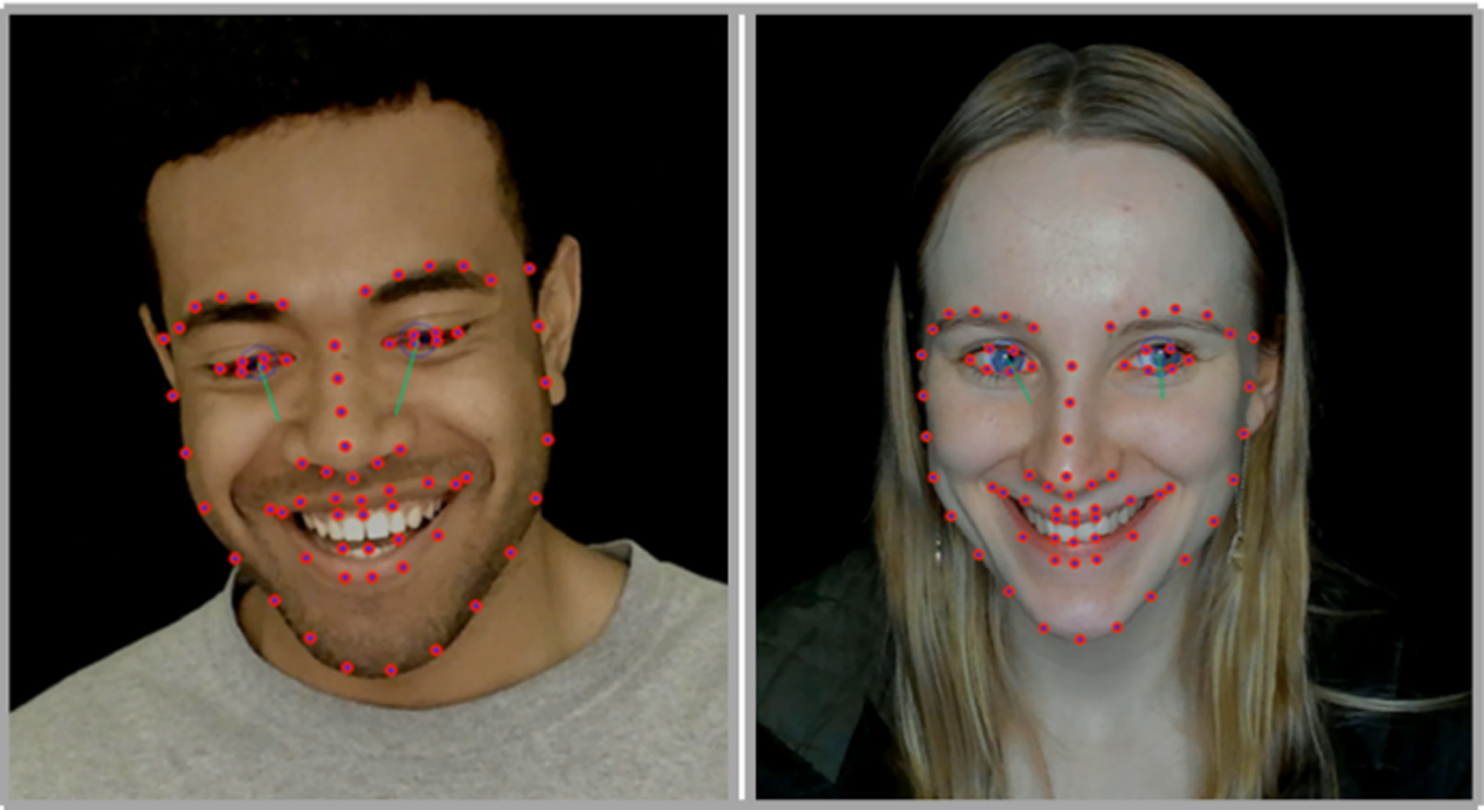
Example frame from smiling study participants, with activation of AU6 > 0.5 and AU12 > 1.5

OpenFace2.2.0 was used as a platform to extract information about facial AUs, which were considered relevant for this study.^21^ This is an open‐source automatic facial recognition software intended to be used by researchers interested in machine learning, affective computing, and facial behaviour analysis.^21^ The software is an update of a previous version of a facial behaviour analysis toolkit, which is based on convolutional neural networks and allows automated identification of 68 facial landmarks at any frame rate.^21^, ^22^, ^23^ The software's output includes a timestamp, quantitative information about all facial landmarks, head posture, eye gaze, activation levels of facial AUs, and three‐dimensional (3D) coordinates of individual facial landmarks, as detected in each frame of the analysed video. AUs represent individual components of muscle movements, whose activation is identified by monitoring the 3D displacement of facial landmarks, with specific sets of landmarks corresponding to each AU. The software also generates videos showing dynamic changes of identified facial landmarks, and 3D information on the gaze vector and the head posture. An example of facial landmarks identified during smiling is shown in Figure 1.

As this study focused on smiling, the relevant AUs were: AU6, AU12, and AU25. AU6 (‘cheek raiser’) tracks the activity of the *orbicularis oculi* muscle, *pars orbitalis*, and is generally considered a marker of smile genuineness (i.e. a Duchenne marker). AU12 (‘lip corner puller’) tracks the activity of the *zygomaticus major* muscle. AU25 (‘lips apart’) tracks the activity of the *depressor labii inferioris* muscle. The intensities of AU6 and AU12 activation were automatically coded by the software using a six‐point ordinal scale (0–5), with values amounting to 1–2 indicating weak (trace) to slight activation, a value of 3 indicating marked pronounced activity, a value of 4 indicating extreme activation, and a value of 5 indicating maximum possible intensity.^15^, ^24^ AU25 was assigned a dichotomous value, which was either 0, indicating lips closed without teeth showing, or 1.

A dedicated software script was developed in Java (Oracle JDK 1.8.0_111). The software had a stand‐alone user‐friendly graphic interface, which allows users to open the output file of OpenFace Software and to detect all the smiling episodes occurring throughout an entire video or within a well‐defined portion of a video, as defined by the start and end frame numbers.

To identify the onset of a smiling episode, both AU6 and AU12 had to be above the specified thresholds. The end of the smiling episode was identified by a subthreshold activation of either AU for longer than 2 s. In effect, this means that when two or more smiling episodes were separated by less than 2 s, they were merged into a single episode. The stand‐by time could be changed by the user.

For every smiling episode, the software assigned a progressive count, the onset time, the duration, and the mean activation of AU6 and AU12 across the entire episode. The onset and duration of individual episodes were given at a resolution equal to the inverse of frame rate of the video analysed.

In order to assign a clinically meaningful value to AU25, this was reported as the proportion of time teeth were shown during a given smiling episode. For example, an activity value of 50% indicated that teeth were visible during half of the episode. Additional outcome measurements included the number of smile episodes per minute and the relative smile time (%). This was calculated as the proportion of time that each individual had smiled while watching the video clip, by summing the durations of all smiling episodes and then dividing the total duration of smiling by the length of the video.

### 2.2 Phase 2: descriptive study and software validation

Data were collected at the Craniofacial Clinical Research Laboratory at the Faculty of Dentistry, University of Otago, under local Ethics Committee approval number H19/160. All participants enrolled in this study agreed to participate and signed a written informed consent form. The report of phase 2 conforms to the guidelines for reporting observational studies (STROBE).^25^

### 2.3 Sample characteristics

A convenience sample of thirty participants (16 females, 14 males; age 18.9 years SD 2.2 years) were recruited as part of a larger project aiming to investigate the impact of oral health, psychological traits, and sociodemographic variables on smiling behaviour. Recruitment started in September 2020 and ended in December 2020.

Participants aged 16–22 years were recruited through local public advertisements, including university mailing lists, social media, flyers, and word of mouth. Exclusion criteria were: (a) cleft lip/ palate or other craniofacial syndromes; (b) severe periodontitis affecting front teeth; (c) history of major psychiatric disorders; (d) Bell's palsy; (e) removable dentures; (f) enamel dysplasia or severe stains affecting front teeth; (g) history of dysmorphophobia. Wearing eyeglasses was not set as an exclusion criterion; however, only one participant requested to wear glasses while watching the videos, and this apparently did not interfere with landmark identification. The sample investigated in this study represented a randomly selected subset of around a hundred study participants. The large sample exhibited a variety of occlusal conditions and is part of another related investigation examining the influence of malocclusion on smiling features through the proposed approach.

The occlusal characteristics of study participants were assessed using the Dental Aesthetic Index (DAI). DAI is a popular tool used in epidemiology to assess a specific set of occlusal traits, such as missing anterior teeth, crowding and spacing in the incisal region, midline diastema, overjet, anterior open bite, incisor irregularity, and molar relationship.^26^ The overall DAI assessment scores of the weighted components are summed with a constant of 13 to produce the final DAI aggregate.

### 2.4 Experimental Setup

An Ultra High‐Definition web camera (Logitech BRIO 4K Ultra High‐Definition Webcam), with resolution set to 4096 × 2160 pixels and frame rate set at 30 frames per second was secured atop a 27‐inch Dell Ultrasharp U2715H computer monitor with a resolution of 2560 × 1440 pixels used to showcase a video clip.

Each participant was seated 60–70 cm away from the display monitor. The height of the monitor was adjusted so that the participant's eyes were aligned at a point corresponding to the middle of the screen when the participant's head was in natural head position.

Face lighting was individually optimised by a ring light (APEXEL 10″ 26 cm LED Selfie Circle Ring, Apexel), which was also secured on the back of the screen. A neutral background was used to avoid light reflections and object interferences, which could affect off‐line analyses of the video. The room light was switched off during the entire recording.

### 2.5 Smile triggering video

Three amusing video clips were identified via a small pilot study by the focus group previously described. The first clip showed an episode of *Mr Bean* (Mr Bean Rides Again, Act 5: The Flight; 3 min), whose character is widely used as a trigger stimulus in smile research.^27^ The next two clips included a non‐stop laughter of a cute baby (47 s) and Juan Joya Borja's viral laughing video widely known as the ‘Spanish laughing guy’ in a televised episode of Ratones Coloraos, which first aired in 2001 but went viral in 2007 (46 s).^28^ The three clips were separated by fade‐outs and merged into a single video, 4 min and 33 s in length (4 min and 24 s without transitions).

Following the amusing video clips, the video presented instructions for completing a series of tasks, with time kept by a countdown timer and progress bar. The tasks involved initiating a series of jaw movements and facial expressions that could confound identification of smiles: speaking (counting 1–10), yawning, coughing, mouth covering; and posing anger, sadness, fear, surprise, disgust, smiling, and neutral expressions. The speaking task lasted 10 s while all other tasks lasted six seconds, with a six‐second inter‐task interval. All tasks were administered once, except for smiling, which was repeated three times. These tasks allowed a precise tuning of the machine learning models that were applied to detect smiling episodes in the video and individual‐specific calibration of the algorithm.

### 2.6 Procedure

Each participant's involvement in the study took place in a single session. At the start of this session, each participant was checked against the inclusion/exclusion criteria, and the occlusal characteristics were scored using the DAI index. The participants were then given an overview of the research project and signed the written consents for participation. To elicit natural responses and trigger spontaneous smiling reactions during the video recording, the participants were not told that the main outcomes of the study were the features of their smiles. Afterwards, each participant was left alone in the recording room and was requested to view the video clip and then perform the follow‐up tasks.

After viewing the video, each participant was asked to fill in two questionnaires. The first was a 12‐item Smile Aesthetics‐Related Quality of Life (SERQoL) questionnaire relating to three dimensions of the psychosocial impact of smiles.^29^ The second was the 60‐item IPIP–NEO–60 personality scale.^30^ The results of these questionnaires were the subject of another investigation and are not analysed in this report. Each participant was given a $20 voucher as reimbursement for participation in this project.

### 2.7 Data analysis and statistics

The full‐face videos were reviewed and coded frame‐wise by two examiners (HM and RK), who were instructed to identify each distinct smiling episode (i.e. preceded and followed by a smile‐free period of at least two seconds) in each study participant. The frames corresponding to the onset and cessation of each smiling episode were identified and noted between the two coders who viewed the full‐face videos within the same setting until a consensus between the two coders was reached. When consensus was not reached, a third coder (MF) was consulted.

The validity of the smiling detection software was assessed by calculating receiver operating characteristics (ROC) curves, using the examiner‐coded smiles as a reference standard and classification variable. ROC curves were assessed frame‐by‐frame timewise for each smile and smile‐free portions. Sensitivity (Se = true positive rate) and specificity (Sp = true negative rate) were calculated frame‐wise and maximised using Youden index (Se +Sp −1). The ROC curve was plotted by false positive rate (1‐Sp) on the x‐axis and the true positive rate (Se) on the y‐axis. The ROC curve was plotted by varying two different thresholds, the first one (Th_1_) representing the activation level of the cheek raiser muscle (AU6) and the second one (Th_2_) representing the activation level of the lip corner puller muscle (AU12). The two thresholds Th_1_ and Th_2_ were varied stepwise using a 0.05 step for both thresholds. The area under the curve (AUC) of the ROC curve and the overall accuracy of the test (sum of true positive frames + true negative frames/total frames) were also calculated.

After threshold optimisation, the software script was run on the entire recording (including the post‐video tasks) to identify smiling episodes and to investigate possible misclassifications (false positives) of confounding tasks as smiles. The three smiling tasks, part of the second section of the video, were excluded from the confounds analysis.

To obtain an estimate of smile genuineness (0–5), intensity (0–5), and teeth exposure (%), the amount of activation of AU6, AU12, and AU25 were averaged across each episode. The outcome variables considered in this study were the number of smiling episodes per session, the mean and cumulative duration of smiling episodes, and the mean activation of AU6, AU12, and AU25.

All the data were analysed in Excel (Version 16.51, Microsoft Corporation) and SPSS (version 20.0 IBM Corporation).

## 3 RESULTS

Study participants were young adults, mostly Caucasian (>80%), about half of them were females, and had a broad range of malocclusions (Table 1).

**TABLE 1:** Demographic characteristics of the participants

Variable	
Age in years [Mean (SD)]	18.9 (2.2)
Gender [*n* (%)]	
Female	16 (53.3)
Male	14 (46.7)
Ethnicity [*n* (%)]	
Caucasian	25 (83.5)
Pacific Islander	2 (6.5)
Other	3 (10.0)
DAI scores [Mean (SD)]	30.7 (9.6)

**FIGURE 2:**
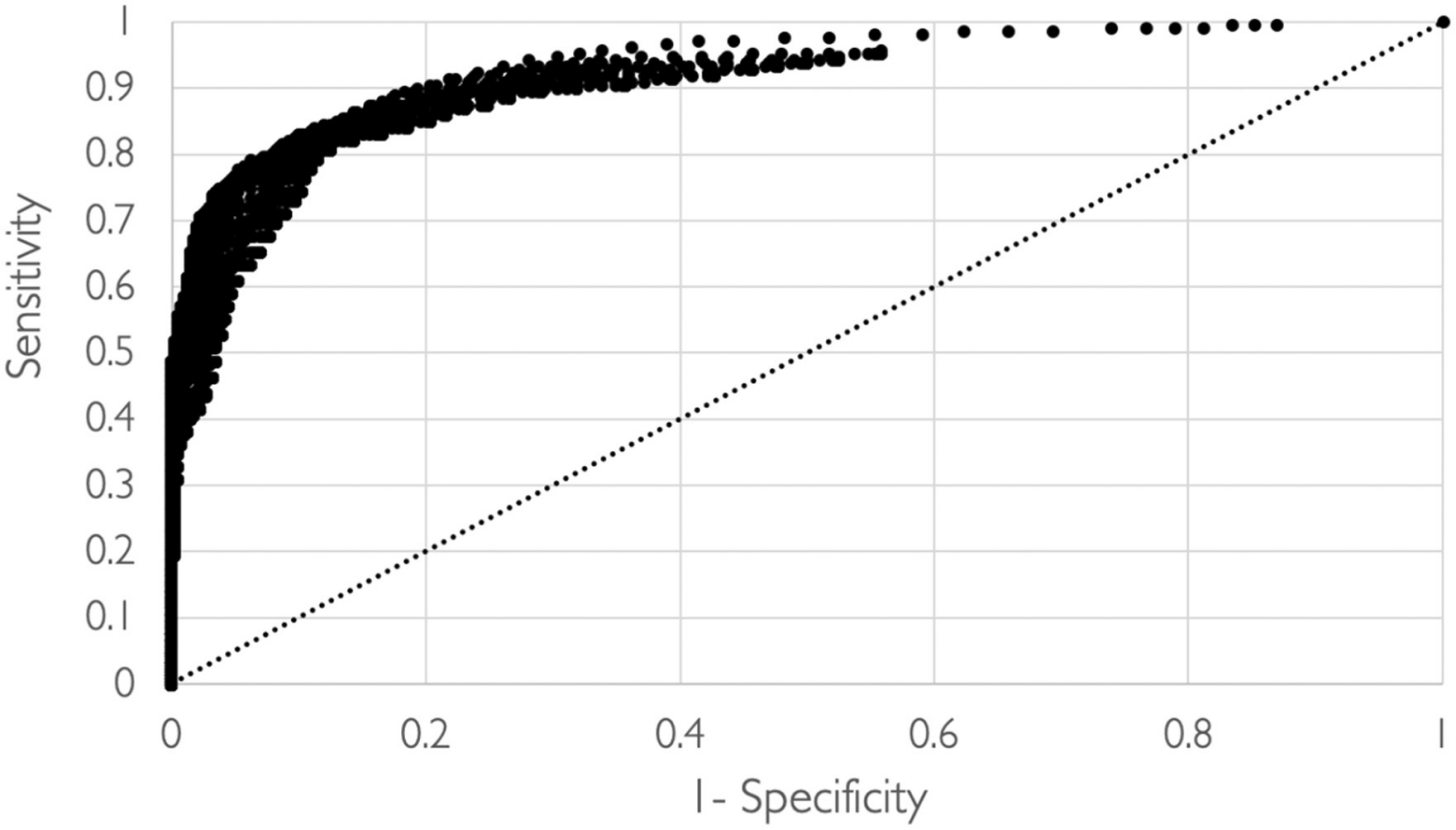
ROC curve based on two thresholds for AU6 and AU12 and frame‐wise detection of smiles

The distinct smile episodes that were manually identified frame‐wise by coders were used to build a ROC curve (Figure 2). The area under the curve of the ROC curve was 0.94, and the overall accuracy of smiling frames detection was 84.5%. The maximisation of Youden index indicated that detection accuracy was highest with thresholds of 0.5 for AU6 and 1.5 for AU12. These thresholds resulted in a sensitivity of 82.9% and a specificity of 89.7% and were used in subsequent episode‐wise analysis in the study sample.

After calibration of the algorithm, the true‐positive detection of individual smiling episodes was 90.0%. In addition, 11.3% of confounder tasks were detected as false positives. The tasks more often misclassified as smiles were mouth covering, which amounted to around one‐third of the false detections and yawning, which amounted to around 20% of the false positive detections.

**FIGURE 3:**
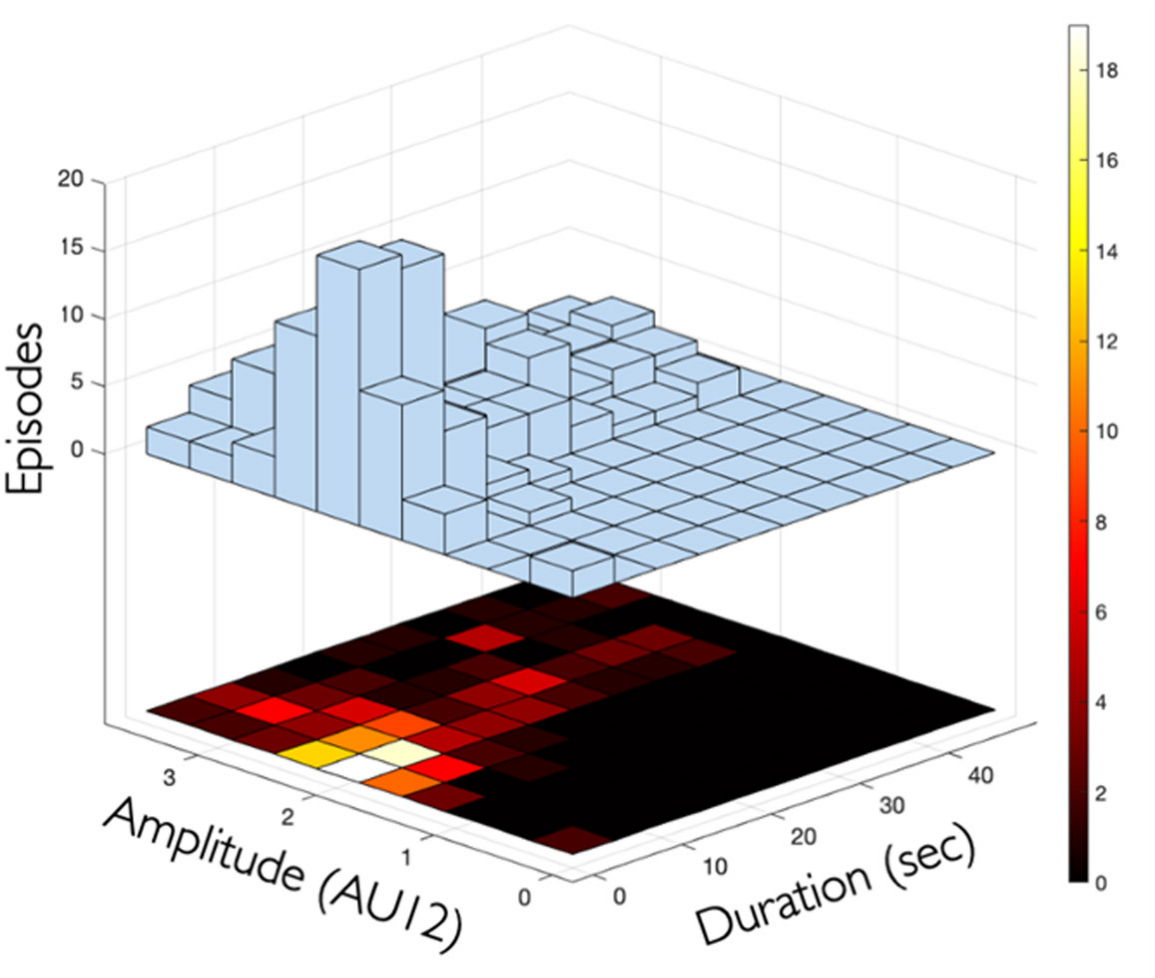
Three‐dimensional histogram depicting all the smiling episodes detected by duration and intensity of AU12.

Study participants smiled approximately seven times according to the classifier, with each smile episode lasting approximately 10 s, or about one‐third of the duration of the humorous videos. The features of smiling episodes showed a large inter‐individual variation in the frequency, intensity, and duration of smiles (Figure 3).

Descriptive statistics for the individual features of smiles, such as activation of specific AUs are given in Table 2. Activation of AU12, which is the main AU of the smile,^15^ ranged from slight to pronounced, with intensity ranking. Some participants hardly showed teeth on smiling, while others showed teeth throughout the entire smile episode.

**TABLE 2:** Descriptive statistics for the features of smiling episodes detected from the 30 study participants, while watching the video footage

	Mean	Standard Deviation	Minimum	Maximum
Number of episodes per minute	1.6	0.8	0.2	3.1
Mean Duration of episode (s)	11.3	5.6	2.3	19.2
Relative Smile Time (%)	34.0	23.5	1.1	80.6
Genuineness (AU6; 0–5)	1.3	0.4	0.4	2.1
Intensity (AU12; 0–5)	2.2	0.4	1.6	3.0
Tooth show (AU25; %)	47.2	27.4	0.8	100

## 4 DISCUSSION

This paper presents a user‐friendly automated software script, which can detect and quantify smiles features in terms of their: (1) frequency, (2) onset and offset of each episode and overall episode duration, (3) peak intensities, (4) percentage of tooth display in each smiling episode and (5) smiling genuineness. In order to identify spontaneous smiles, we have introduced two detection thresholds based on the levels of activation of the cheek raiser (AU6, a Duchenne marker) and the lip corner puller (AU12). The findings indicate an acceptable detection accuracy of the proposed method, which can be used for an episode‐wise analysis of smiles. The software does not require calibration and is available upon request.

To the best of our knowledge, this is the first study that has used available libraries and FACS to present an automated episode‐wise analysis of smile episodes. This script uses a user‐friendly interface, which needs to be used in unison with OpenFace2.20 (an open‐source script), which any researcher can utilise. Moreover, the introduction of Artificial Intelligence (AI) to the field of dynamic smile analyses is fundamental. Hence, observer‐related biases are minimised with an expected reduction in time and labour associated with manual analyses.^31^

The measure of diagnostic accuracy includes both sensitivity and specificity values.^32^ In our study, 82.9% sensitivity value demonstrates a high proportion of detected true smile episodes. In addition, the diagnostic specificity is confined to 89.7%, as presented in the ROC curve plot. In summation, both values align well with stipulated expectations in the area of automated facial expression recognition and dynamic analysis of human emotions.^33^, ^34^ On the other hand, descriptive values from automated analysis of sample clips showed that participants smiled around two times per minute, on average for around 11 s per episode. Also, the mean intensity of the *zygomaticus major* activation (AU12) was 2.2 ± 0.4. These findings align with previous research of participants who viewed a funny clip with a mean duration of AU12 activation (13.8 ± 12.7 s) and a maximum intensity of 1.8 ± 1.1.^35^ Further, a recent study reported that the mean intensity of AU12 was 4.1 during genuine smiling and 3.9 for posed smiles.^36^ Though these AU12 intensity values seem comparable during both genuine and posed smiles, previous research pointed out to recognisable differences with respect to AU6 activity during genuine and posed smiles placing an argument that it would be difficult to deliberately fake a genuine smile.^10^, ^37^ However, there is also some evidence suggesting that Duchenne smiles are merely traces of smile intensity rather than a reliable and distinct indicator of smile authenticity.^38^ Such discrepancies in the reported findings may be ascribed to the different methods to trigger and to measure smiles, the social context, and sociodemographic characteristics of the sample, which may all influence the features of smiling.^39^ However, they still do impose limitations on our understanding, recognition and differentiation of genuine and posed smiles.

Smiling is an expression that can be triggered on demand, as well as spontaneously within a social context. In phase 2, the trigger video had successfully elicited smiles in individuals regardless of their ethnic background, age, or malocclusion. This suggests that individuals are prone to smiling when a suitable trigger is used aside from circumstances and situations where social integration is seen.^40^ In addition, while the participants were informed about the video recording process, it can be argued that masking the purpose of the video recording being the assessment of smiling episodes succeeded in rendering a natural response to the trigger (i.e. spontaneous smiling) as observation awareness is well‐known to be an important variable in smiling research.^39^

It is possible to detect various spontaneous facial expressions expressed in unscripted social context with automated recognition systems.^41^ However, establishing reliable automated coding of discrete facial expressions is a challenging process.^41^ Detection issues often arise when multiple AUs are involved; hence, recognising compound facial expressions where individuals combine various expressions, is daunting.^42^ In addition, dynamic tracking and head orientation also pose obstacles to AI recognition.^43^ In our study, we incorporated post‐video scripts of different plausible confounders to tackle these issues. These tasks included: counting numbers, yawning, coughing, mouth covering; and posing anger, sadness, fear, surprise, disgust, and neutral expressions.^44^ Our findings show that only 11.3% of the tasks were identified as false positives after calibration of the algorithm. Misclassified smiles were mostly associated with mouth covering and yawning. In turn, the proposed method for episode‐wise detection of smiles could serve as a cornerstone to better landmark feature extraction as well as recognition detection in future research.^45^

The present study has some limitations, which should be emphasised. Most obviously, the software was validated on a relatively small convenience sample of mostly Caucasian adolescents and young adults who spontaneously smiled. Further research is needed to investigate the quantitative features of posed and spontaneous smiles and validation of the proposed method in other samples, which have not been analysed in the present study. Also, research is needed to determine the classifiers' accuracy in larger and more heterogeneous sample to investigate the effect of ethnicity, sex, age and other demographic characteristics on smiling and to increase the external validity of our findings.^46^ In addition, it is important to note that the yielded accuracy was not perfect, though an AUC value closer to 1 (0.94 in our report) is viewed as very high in terms of the discrimination performance of the software.^47^ Further enhancements are plausible with future improvements in AI. These enhancements should also include other methods of quantification of muscular activity to objectively assess genuine smiles using electromyography (EMG) and wearable detection devices.^48^ The possible effect of calibration on smile detection accuracy also needs to be further investigated in different samples and in contrast with other smile detection methods to establish which is better in terms of accuracy, cost, labour, and overall handling.

In summary, this paper presents a novel automated episode‐wise quantitative assessment of smiling dynamics. The software can provide a quantitative analysis of the frequency, duration, intensity, and characteristics of smiles through a user‐friendly interface that is available for further utilisation in smile research. The proposed approach has potential different applications within dentistry. Firstly, it would offer researchers the opportunity to understand to what extent oral conditions, such as dental, oral, and facial anomalies objectively influence smiles. This could then translate into investigations of the effect of corrective treatment through oral rehabilitation, dental and orthodontic treatment, on specific features of smiles. Furthermore, the proposed approach may be used to trigger and investigate spontaneous smiles, thus facilitating restorative treatment plans, as compared to information depicted via static posed photographs.

In addition, the capability of the algorithm to detect and analyse observable data of individuals smiling under controlled conditions opens the door to addressing further challenges in other areas. For example, future research could target understanding the dynamics of smiling in real‐time conditions. Moreover, the dental literature is replete with research on the static features of smiles, while data are scarcely available on the dynamic features. It would be interesting and important to examine the relationship between malocclusion patterns, different orthodontic treatment modalities, and their effect on smiling from a dynamic standpoint. Based on the aforementioned points, future developments, and further implementation of the pattern‐recognition algorithm could be significant not only in dental disciplines, but also in psychology, sociology, and behavioural research.

## 5 CONCLUSIONS

Individual smile episodes and their quantitative features, such as frequency, duration, genuineness, and intensity can be automatedly assessed with an acceptable level of accuracy. The proposed approach can be used to investigate the impact of oral health and oral rehabilitation on smiles.

## AUTHOR CONTRIBUTIONS

Hisham Mohammed and Reginald Kumar Jr involved in conceptualisation, investigation, validation and writing the original draft. Hamza Bennani involved in software and analysis. Jamin Halberstadt involved in methodology, reviewing and editing and supervision. Mauro Farella involved in conceptualisation, methodology, reviewing and editing, analysis and supervision.

## CONFLICT OF INTEREST

All authors declare that they have no competing interest.

### PEER REVIEW

The peer review history for this article is available at https://publons.com/publon/10.1111/joor.13378.

## Data Availability

The data would be available upon reasonable request.
